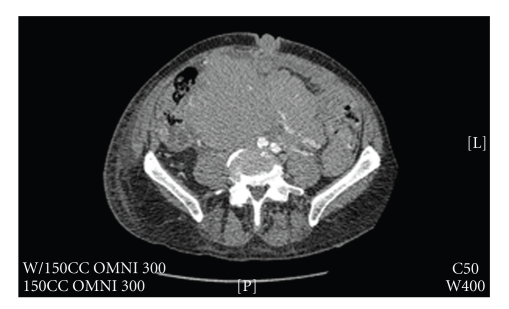# Relapsed Mantle Cell Lymphoma Presenting as “Sister Mary Joseph Nodule”

**DOI:** 10.1155/2010/708348

**Published:** 2010-04-08

**Authors:** Jennifer E. Vaughn, Ajay K. Gopal

**Affiliations:** ^1^Department of Internal Medicine, University of Washington, Box 39892. 325 Ninth Ave., Seattle, WA 98104, USA; ^2^Clinical Research Division, Fred Hutchinson Research Center, Seattle, WA, USA; ^3^Seattle Cancer Care Alliance, Mailstop G6-800, 825 Eastlake Ave. E Seattle, WA 98109, USA

## Abstract

The finding of umbilical metastasis has historically been called a “Sister Mary Joseph Nodule”. A few case reports of lymphoma presenting in this manner have been documented. We report a case of relapsed mantle cell lymphoma (MCL) presenting as a Sister Mary Joseph's nodule. Although the few reports of lymphoma exhibiting umbilical metastasis suggest that patients may still expect a reasonable response to chemotherapy, this patient experienced multiple relapses, despite aggressive chemotherapy regimens. This clinical course is characteristic of the mantle cell form of non-hodgkin's lymphoma and illustrates a need to seek out more effective therapies.

## 1. Introduction

Umbilical malignant metastasis is a rare sign of advanced malignancy and usually associated with cancers of the gastrointestinal and reproductive tract [1]. The term “Sister Mary Joseph's nodule” was coined by Sir Hamilton Bailey, after Sister Mary Joseph Dempsey (1856–1939) who served as surgical assistant to Dr. William Mayo in Rochester, MN. She was the first to observe the association between umbilical nodules, which she named “pants button umbilicus,” and metastatic intraabdominal disease. This discovery was published in 1928 [2]. As was also noted by its namesake, it is generally thought to signify a poor prognosis, with mean survival reported between 8 and 11 months [1, 3, 4].

Interestingly, reports of hematological malignancies presenting in this manner are exceedingly uncommon and when reported, do not necessarily represent intractable disease. In a review of 407 cases of SMJ nodules, only 1 was noted to be of lymphomatous origin [5, 6]. Further review of the literature revealed five cases of SJM nodules determined to be from metastatic lymphoma [5–10] with four demonstrating significant responses to chemotherapy [7–10] and two [7, 8] achieving complete remission. All five case reports identified the malignancy as non-Hodgkin lymphoma and two of these were specifically identified as large B-cell lymphomas [8, 9]. None were of MCL origin. In fact, a dedicated search revealed no cases of MCL presenting as SMJ nodule in the literature.

## 2. Case Report

A 72-year-old male with a history of previously treated blastic variant (Ki-67 60%–70%) mantle cell lymphoma (MCL) presented to his outpatient oncology clinic with new complaints consisting of diffuse abdominal pain and distention, night sweats, fatigue, and a new mass within the umbilicus. The patient was noted to have a Mantle Cell Lymphoma International Prognostic Index (MIPI) [11] of 6, primarily receiving a high risk score due to age >70 and an LDH which was 1.4 times the upper limit of normal. The patient's performance status was limited only by weakness attributed to chronic narcotic use, but he was otherwise very functional at the time of presentation.

**Figure 1:**
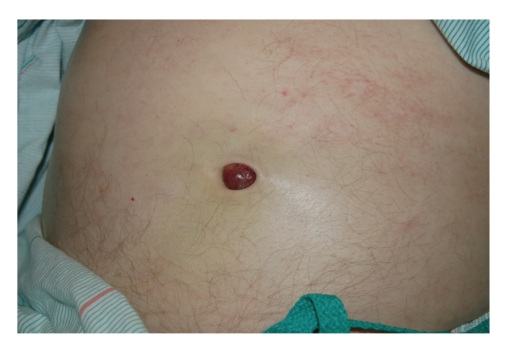


 Recurrence was noted by PET scan to have occurred in areas of previous involvement, including several areas above the diaphragm, the retroperitoneum, and the right pelvis. However, abdominal examination revealed a new, nontender, violaceous mass projecting from the umbilicus (Figure 1). Computerized tomography of the chest, abdomen and pelvis confirmed the presence of the lesion within the umbilical cavity and also demonstrated an abdominal mass measuring 20.7 cm × 30 cm × 13 cm abutting the stomach against the diaphragm (Figure 2). This constellation of findings was thought to represent relapsed MCL and the protuberant abdominal mass consistent with the finding of a Sister Mary Joseph's (SMJ) nodule. The patient experienced multiple relapses after R-CHOP, single agent rituximab, bortezomib, and gemcitabine-carboplatin-dexamethsone-rituximab. Following this most recent relapse, he experienced a transient response to cyclophosphamide, etoposide, dexamethasone, and rituximab, but unfortunately expired 11 months later after further tumor progression.

## 3. Discussion

MCL is characterized by frequent extranodal involvement with a predilection to the aerodigestive tract, though ocular, central nervous system, breast, skin, and testicular involvement have been reported [12–17]. Studies meant to characterize the frequency of spread to these sites are often limited by low case numbers and methodology of patient selection. Clinically significant gastrointestinal involvement has been historically reported in 15%–30% of cases [18–21] although studies of random biopsy specimens from the GI tracts of untreated MCL patients suggest that this number may much higher [18, 22]. Central nervous system (CNS) infiltration has been described in anywhere of 2%–23% of MCL patients[15, 23, 24] and was associated with high tumor proliferative rate, blastic variant, and elevated lactate dehydrogenase [25]. Factors associated with other sites of extranodal disease beyond the gastrointestinal tract, bone marrow, and CNS are less well described but are also likely correlated with these same factors.

In general, MCL is characterized by lack of sustained responses to conventional therapies and poorer outcomes when compared to other lymphomas. While five-year survival after standard R-CHOP therapy was recently demonstrated to have improved from 22% to 47% [26], worse outcomes are associated with the presence of >1 entranodal site of disease [20, 27]. This case presentation illustrates both another unusual extranodal manifestation of MCL and the still grave prognosis of this entity emphasizing the need for more effective treatments.

## Figures and Tables

**Figure 2:**